# Differences between Dorsal and Ventral Striatum in the Sensitivity of Tonically Active Neurons to Rewarding Events

**DOI:** 10.3389/fnsys.2017.00052

**Published:** 2017-07-24

**Authors:** Kevin Marche, Anne-Caroline Martel, Paul Apicella

**Affiliations:** Institut de Neurosciences de la Timone UMR 7289, Aix Marseille Université Marseille, France

**Keywords:** basal ganglia, motivation, prediction, learning, primate

## Abstract

Within the striatum, cholinergic interneurons, electrophysiologically identified as tonically active neurons (TANs), represent a relatively homogeneous group in terms of their functional properties. They display typical pause in tonic firing in response to rewarding events which are of crucial importance for reinforcement learning. These responses are uniformly distributed throughout the dorsal striatum (i.e., motor and associative striatum), but it is unknown, at least in monkeys, whether differences in the modulation of TAN activity exist in the ventral striatum (i.e., limbic striatum), a region specialized for processing of motivational information. To address this issue, we examined the activity of dorsal and ventral TANs in two monkeys trained on a Pavlovian conditioning task in which a visual stimulus preceded the delivery of liquid reward by a fixed time interval. We found that the proportion of TANs responding to the stimulus predictive of reward did not vary significantly across regions (58%–80%), whereas the fraction of TANs responding to reward was higher in the limbic striatum (100%) compared to the motor (65%) and associative striatum (52%). By examining TAN modulation at the level of both the population and the individual neurons, we showed that the duration of pause responses to the stimulus and reward was longer in the ventral than in the dorsal striatal regions. Also, the magnitude of the pause was greater in ventral than dorsal striatum for the stimulus predictive of reward but not for the reward itself. We found similar region-specific differences in pause response duration to the stimulus when the timing of reward was less predictable (fixed replaced by variable time interval). Regional variations in the duration and magnitude of the pause response were transferred from the stimulus to reward when reward was delivered in the absence of any predictive stimulus. It therefore appears that ventral TANs exhibit stronger responses to rewarding stimuli, compared to dorsal TANs. The high proportion of responsive neurons, combined with particular response features, support the notion that the ventral TAN system can be driven by specific synaptic inputs arising from afferent sources distinct from those targeting the dorsal TAN system.

## Introduction

Tonically active neurons (TANs) constitute a group of neurons in the striatum that are readily identified by their spiking characteristics in electrophysiological studies performed in behaving animals (Apicella, 2002). There is broad acceptance that they correspond to cholinergic interneurons which comprise less than 3% of the total striatal cell population (Tepper and Bolam, 2004; Kreitzer, 2009). The disruption of the intrinsic cholinergic innervation of the striatum has been linked to brain disorders, including motor and psychiatric abnormalities (Pisani et al., 2007; Deffains and Bergman, 2015). Although cholinergic interneurons are sparsely distributed, they densely innervate the striatum and act locally to modulate striatal output (Zhou et al., 2002; Pisani et al., 2007). Cholinergic transmission is also known to be directly involved in the modulation of striatal dopamine (DA) release (Threlfell and Cragg, 2011; Cachope et al., 2012; Threlfell et al., 2012) and both systems have been implicated in the regulation of striatal synaptic plasticity (Kreitzer and Malenka, 2008; Di Filippo et al., 2009; Surmeier et al., 2009).

Behaving monkey experiments have led to the prevailing view that TANs carry signals that are important for implementing reward-guided learning (Aosaki et al., 1994; Apicella et al., 1997; Ravel et al., 2003; Morris et al., 2004; Adler et al., 2013). In this regard, responses of TANs and midbrain DA neurons to motivationally salient events are quite similar (Apicella, 2007), notwithstanding differences in the polarity of their modulation. There has been interest in comparing their sensitivity to errors in the prediction of reward (i.e., the difference between the outcome of a situation or action and its prediction) which are of crucial importance for the formation of stimulus–reward associations (Sutton and Barto, 1998; Schultz, 2002). It was shown that TANs provide a signal that reports whether the rewarding outcome deviates from what is expected. In particular, TAN modulations at reward delivery reflect the probability of reward, becoming stronger with decreasing reward probability (Joshua et al., 2008; Apicella et al., 2009, 2011). Also, some TANs display a decrease in activity after reward delivery and an increase in activity when reward is omitted, possibly reflecting differential coding of the presence and absence of upcoming reward (Apicella et al., 2009). These TAN modulations have been evidenced in dorsal regions of the monkey striatum. A study in behaving rats has shown that a large proportion of putative TANs located in the ventromedial part of the striatum respond to unexpected reward delivery and omission by an increase and decrease in firing, respectively (Atallah et al., 2014). As reported by the same research group, some TANs in dorsal parts of the striatum display changes in activity when rewards are delivered on correct performance (Thorn and Graybiel, 2014), but it is uncertain whether they may display bidirectional outcome signaling in a manner similar to that described in the ventromedial striatum. Another study has indicated that TANs in both dorsolateral and ventral striatum respond to reward delivery, but do not appear to be sensitive to reward omission (Benhamou et al., 2014). However, it cannot be excluded that the group identified as TANs in the rodent striatum may contain some non-cholinergic neurons (Beatty et al., 2012) exhibiting distinct response profiles during task performance.

Different subdivisions of the striatum are assumed to subserve distinct roles in learning. The dorsal striatum mediates flexible action selection and automatic action, whereas the ventral striatum is thought to be critical for learning the value of stimuli, irrespective of the action selected in response to these stimuli (Balleine et al., 2009). Also, an alteration in cholinergic innervation of the ventral striatum has been reported in Alzheimer’s disease (Lehéricy et al., 1989) and schizophrenia (Holt et al., 2005). Inactivation studies on rodents have pointed to the involvement of the cholinergic system of the ventral striatum in mood control and emotional behavior (Witten et al., 2010; Warner-Schmidt et al., 2012). Until now, studies examining TAN activity in behaving monkeys have emphasized that TAN responses to rewarding events occur throughout the mediolateral extent of the dorsal striatum, encompassing regions that are more closely tied to the motor and cognitive aspects of task performance. Only one study reported a variation of response properties of TANs that may be related to the regional specializations of the dorsal striatum (Yamada et al., 2004) with TAN responses to a stimulus that triggers movement being more frequent in the posterior putamen (i.e., motor striatum) than in the caudate nucleus (i.e., associative striatum). However, no monkey study has yet monitored TAN activity in the ventral striatum, including the nucleus accumbens, which is specialized for processing of affective and motivational relevance of stimuli (Haber and McFarland, 1999). It is therefore an open question whether responses differ between TANs in the dorsal striatum and those in the ventral striatum.

Here, we examined TAN activity under the same behavioral conditions and compared their responsiveness to rewarding events according to their location in dorsal or ventral parts of the striatum. We found that the TANs sampled all displayed, on average, stronger responses to rewarding events in ventral than dorsal striatum, with specific response features occurring in the ventral striatum. These findings indicate that TAN signaling may rely on distinct afferent inputs to striatal cholinergic interneurons.

## Materials and Methods

**Figure 1:**
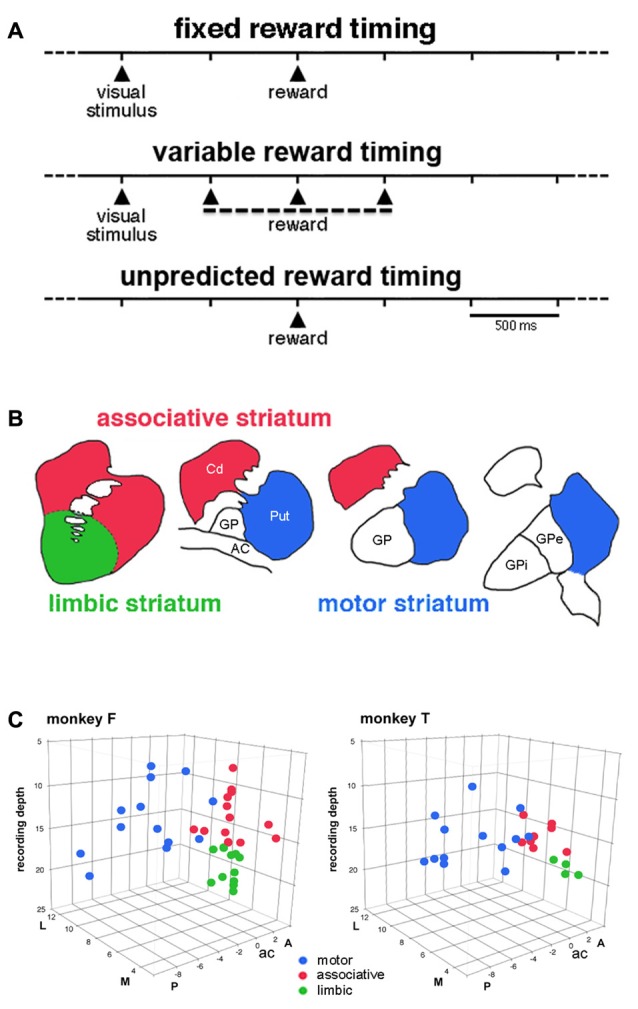
Timing of events in the testing conditions and approximate functional subdivisions of the striatum. **(A)** Temporal structure of the sequence of events in the different testing conditions. We used a classical conditioning task in which the presentation of a visual stimulus preceded the delivery of a liquid reward with a constant time interval of 1 s, in the fixed reward timing (FRT) condition, or a pseudorandomly varying interval of 1 ± 0.5 s, in the variable reward timing (VRT) condition. In both conditions, the visual stimulus was extinguished 0.3 s after it came up. In the unpredicted reward timing (URT) condition, the liquid was automatically delivered without preceding stimulus. The three conditions were run in separate blocks of trials and the change in condition was not indicated by any external cues. **(B)** A drawing illustrating the tripartite subdivision of the primate striatum in the coronal plane, based on previous studies of the topography of the corticostriatal projection in the macaque monkey (Parent and Hazrati, 1995; Haber and McFarland, 1999). **(C)** Locations of recorded tonically active neurons (TANs) plotted separately for each monkey. Each color dot represents a single neuron. TANs were recorded between 2 mm anterior (A) and 8 mm posterior (P) to the anterior commissure (ac), over the lateral (L) and medial (M) extent of the striatum measured from the midline (in mm). The recording depth (in mm) was determined from a zero reference point as determined by using microdrive readings. The average depth of penetration of 17 mm was used to divide the precommissural striatum into dorsal and ventral regions.

Two adult male macaque monkeys (*Macaca mulatta*), monkeys F and T, were used in the present experiments. All experimental procedures were in compliance with the National Institutes of Health’s Guide for the Care and Use of Laboratory Animals and approved by the Comité d’éthique en Neurosciences INT-Marseille (Protocol A2-10-12). The monkeys were seated in a restraining box facing a vertical panel containing light-emitting diodes (LEDs). Before the recording experiments started, both animals were trained in a probabilistic decision task involving reaching arm movements under probabilistic schedules of reinforcement. Throughout the several months of recording in the striatum, blocks of trials of the instrumental task were interspersed occasionally with a Pavlovian conditioning task in which the delivery of reward was not contingent on a particular behavioral response. In this latter condition, monkeys were passively exposed to a visual stimulus (illumination of the central LED for 0.3 s) automatically followed by the delivery of reward. They were three testing procedures illustrated in Figure 1A: (1) *Pavlovian conditioning with constant time interval*. In this condition, the visual stimulus was followed after a fixed interval of 1 s by the delivery of reward. The onset of the visual stimulus was initiated by the experimenter and occurred at irregular times (>6 s). This condition was designated “fixed reward timing” condition (FRT); (2) *Pavlovian conditioning with variable time interval*. In this condition, the time of reward was semirandomly varied relative to the onset of the visual stimulus by changing the length of the stimulus-reward interval (0.5, 1, or 1.5 s). We refer to this condition as the “variable reward timing” condition (VRT); and (3) *Free reward delivery*. In this condition, the liquid reward was delivered in the absence of any predictive stimulus and with a variable intertrial interval (>6 s) so that its timing could not be accurately predicted by the monkey. We refer to this condition as the “unpredicted reward timing” condition (URT). The three conditions alternated in blocks of 30–40 successive trials and the block transitions were not explicitly signaled. In all conditions, trials lasted 6 s, so that the overall temporal structure of the testing condition and the total number of rewards delivered in each trial block remained the same.

Surgery was carried out under gas anesthesia (isoflurane 2.5%) and aseptic conditions. We implanted a head-holding device and a recording chamber aimed to the striatum. Antibiotics and analgesics were administered after surgery. We recorded the extracellular activity of single neurons with movable glass-coated tungsten electrodes over a wide area of the anterior and posterior regions of the striatum. Electrodes were passed inside a stainless steel guide tube (0.6 mm outer diameter) at the beginning of each recording session. After penetration of the dura, the electrode was advanced toward the striatum with a manual hydraulic microdrive (MO95; Narishige, Tokyo, Japan) until the activity of a single neuron was isolated. Neuronal signals were amplified (×2000), filtered with a band pass of 0.3–1.5 kHz, and converted to digital pulses through a window discriminator (NeuroLog; Digitimer, Hertfordshire, UK). Continuous monitoring of the spike waveform on a digital oscilloscope during recording allowed us to check the quality of spike isolation. Presentation of visual stimuli, delivery of reward and collection of neuronal data for off-line analyses were controlled by a computer, using custom software developed by E. Legallet (LabVIEW, National Instruments, Dallas, TX, USA). Once a neuron was isolated, we usually first examined its activity in the FRT condition. The neuron was then tested in another condition, either the VRT and/or URT condition. The task relationships of neuronal discharges were assessed on-line in the forms of rasters aligned on each task event. The distinctive spontaneous discharge rate and spike waveform of TANs permitted them to be easily identified in extracellular recordings from the striatum of behaving monkeys (Apicella, 2002).

We analyzed neuronal activity by detecting changes in TAN firing on the basis of a Wilcoxon signed-rank test (Apicella et al., 2009). The baseline firing rate was calculated during the 500 ms preceding the presentation of the visual stimulus (FRT and VRT conditions) or reward (URT condition), defined as the control period. A test window of 100 ms duration was moved in steps of 10 ms, starting at the presentation of the visual stimulus or reward delivery. For each time step, we calculated the average spike counts within the interval across all trials. The mean discharge rate in the 100-ms window was compared, at each step, with that in the control period. The onset of a modulation was taken to be the beginning of the first of at least five consecutive steps showing a significant difference (*P* < 0.01, Wilcoxon’s test) as against the baseline activity. The offset of a modulation was defined in the same manner by the first of at least five consecutive steps with activity back to control. We then determined the onset and offset of the modulation in the firing of the neuron with 10 ms resolution. Magnitude of change in TAN activity was assessed by counting spikes between onset and offset of responses for each neuron showing a significant change in firing and expressed as percentage below or above baseline activity.

To assess the properties of the population of TANs sampled in the different conditions, we calculated the ensemble average activity aligned with stimulus onset and reward delivery of all neurons recorded, regardless of their individual responsiveness to events. We computed the average firing rates in 10 ms bins to identify when the population significantly changed its activity, relative to the control period (500 ms before the first task event). We considered the onset time of a change as the first of at least three consecutive bins for which a significant difference was detected (paired *t*-test, *P* < 0.05). End time was defined in a similar manner for the return of the ensemble average activity to that in the control period. Then durations of activity change over the population of neurons were defined on the basis of onset and offset times of these changes.

Differences in fractions of responding TANs among the testing conditions were tested with the chi-square test. Differences in latencies and magnitudes of TAN responses were assessed with the Kruskal-Wallis test (*P* < 0.05), with striatal region as a factor. *Post hoc* comparisons between regions were determined using Wilcoxon’s test. It should be underlined that neuronal data collected in two animals face us with potential problems related to repeated measures taken from the same subjects, thus leading to lack of statistical independence in the data (the so-called “pseudoreplication”, for a review see Lazic, 2010). Such a limitation is inherent in analyses of neuronal data in behaving animals, particularly in monkey studies which typically involve a small number of subjects (*n* = 2). Since we cannot, practically, include a large number of animals in order to satisfy the independence assumption, it should be kept in mind that we rely on standard practices in the field to make inferences about the statistical significance of our data.

In addition to the quantitative assessment of task-related activities of individual TANs, we gave a description of the properties of the population of TANs by pooling activities across samples of neurons recorded in the different striatal regions. For each neuron, a normalized perievent time histogram was obtained by dividing the content of each bin by the number of trials and the population activity was obtained by averaging all normalized histograms referenced to a particular task event. Data were analyzed using custom-written MATLAB (version R2015b, Mathworks, Natick, MA, USA) scripts and statistical comparisons were conducted with JMP software (version 11, Chicago, IL, USA).

Based on the known topographic organization of projections from the cerebral cortex and limbic system, the striatum explored in the present study was divided into a motor region, which corresponds to the part of the putamen posterior to the anterior commissure and an associative region, which predominantly includes the dorsal precommissural parts of both the caudate nucleus and putamen (Parent and Hazrati, 1995). The ventral part of the caudate nucleus and putamen rostral to the anterior commissure is traditionally regarded as the limbic striatum (Haber and McFarland, 1999). The approximate boundary that divides the striatum into, on the one hand, the motor striatum and, on the other hand, the associative and limbic striatum can be determined from the position of the anterior commissure. Because no reliable anatomical or electrophysiological marker could help in defining the border between the dorsal and ventral parts of the precommissural striatum, we used recording depths to tentatively define a boundary between these two subdivisions which was situated at 17 mm which corresponds roughly to 10 mm from the dorsal border of the striatum (Marche and Apicella, 2017).

## Results

We classified recorded striatal neurons into TANs on the basis of well-established electrophysiological characteristics, including tonic irregular spontaneous firing rates, long-duration spike waveforms, and changes in activity during task performance expressed as brief decreases in firing (the so-called “pause”) in response to rewarding events (Kimura et al., 1984; Aosaki et al., 1994; Apicella et al., 1997). We recorded the activity of 62 TANs (monkey F, 37; monkey T, 25) while the animals waited for the reward delivered at the end of the 1-s interval after the onset of the visual stimulus (i.e., the FRT condition). We divided the striatum into three regions which correspond roughly to the functional territories conventionally defined in the primate striatum (Figure 1B). As shown in Figure 1C, 26 neurons were located in the motor striatum (13 and 13 in monkeys F and T), 21 in the associative striatum (13 and 8 in monkeys F and T) and 15 in the limbic striatum (11 and 4 in monkeys F and T). As stated in the “Materials and Methods” Section, the boundary between the associative and limbic striatum was based primarily on recording depths and it can be confirmed that ventral TANs were located in the deepest part of the precommisural striatum (Figure 1C). The mean firing frequency of TANs was similar in all three regions (motor: 5.55 ± 1.59 spikes/s, *n* = 26; associative: 5.41 ± 1.12 spikes/s, *n* = 21; limbic: 5.23 ± 1.13 spikes/s, *n* = 15). We evaluated their responsiveness to either the visual stimulus or reward in terms of a pause in TAN firing, with or without subsequent rebound activation, a very few neurons displaying a brief increase in firing before the pause.

**Figure 2:**
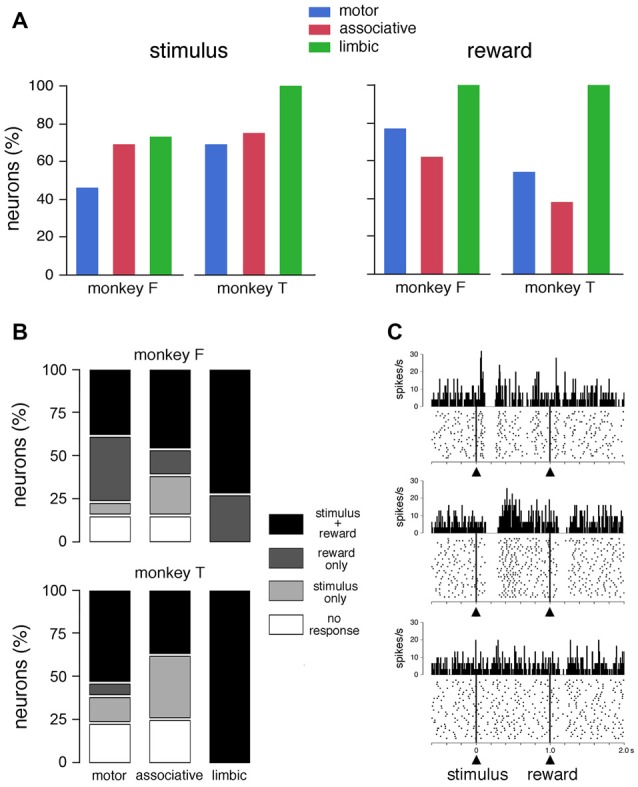
Responses of TANs to task events in the FRT condition. **(A)** Proportions of TANs responding to the visual stimulus and reward. Bar plots for each monkey show proportions of responses across the three striatal regions. **(B)** Relative proportions of TANs showing selective and nonselective responses to stimulus and/or reward. **(C)** Examples of the responses of TANs to the visual stimulus and/or reward. In each panel, a dot represents a neuronal impulse, and a line of dots represents the neuronal activity recorded during a trial. Histograms and dot displays of TAN activity are aligned on the onset of the stimulus. Vertical calibration on histograms is in spikes per second. Bin width for histograms is 10 ms. The data were taken from monkey T (motor striatum, top panel) and monkey F (ventral striatum, middle and bottom panels).

Of the 62 neurons whose activity was recorded in the FRT condition, 42 (68%) showed statistically significant changes in activity after the presentation of the visual stimulus (23 and 19 neurons in monkeys F and T, respectively) and 43 (69%) after the delivery of reward (29 and 14 neurons in monkeys F and T, respectively). The fraction of responsive TANs did not differ significantly between the stimulus and reward in monkeys F (*χ*^2^ = 2.35, *P* > 0.05, chi-square test > 0.05) and T (*χ*^2^ = 2.25, *P* > 0.05). Figure 2A summarizes the percentage of TANs responding to the visual stimulus and reward in each striatal region, separately for each monkey. The proportion of TANs displaying a response to the visual stimulus did not vary significantly among striatal regions (monkey F: *χ*^2^ = 2.19, *P* > 0.05; monkey F: *χ*^2^ = 2.50, *P* > 0.05). The percentage of TANs responsive to reward showed some variation across regions, with a significantly higher proportion of responses in the limbic striatum than in the two other regions in monkey F (*χ*^2^ = 7.26, *P* < 0.05), and a trend in the same direction in monkey T (*χ*^2^ = 5.76, *P* = 0.055). Remarkably, neurons recorded in the limbic striatum (11 and 4 in monkeys F and T, respectively) were invariably responsive to reward, indicating that the TAN sensitivity to reward is the strongest in this region. By pooling the data from the two monkeys, we found that the distribution of TANs responding to reward varied significantly among the three striatal regions (65, 52 and 100%, in the motor, associative and limbic striatum, respectively, *χ*^2^ = 13.80, *P* < 0.01), whereas the distribution of TANs responding to the stimulus did not change significantly (58, 71 and 80%, in the motor, associative and limbic striatum, respectively, *χ*^2^ = 2.40, *P* > 0.05).

Figure 2B shows the relative proportions of the selective and nonselective response types in the three striatal regions for each animal. Of the total of 62 neurons tested in the FRT condition, 32 neurons were responsive both to the stimulus and reward and 21 responded to only one event (stimulus: *n* = 10; reward: *n* = 11). It is notable that the proportion of TANs responding to both stimulus and reward was increased in the limbic striatum compared to motor and associative striatum. On the other hand, responses that were specifically related to the stimulus were not found in the limbic striatum. Examples of TANs with nonselective or selective responses are shown in Figure 2C. The neuron shown in the top panel displayed a modulation of activity after the visual stimulus, with an excitatory component immediately before the pause-rebound response, but its discharge was not influenced by the subsequent delivery of reward. Conversely, the neuron illustrated in the middle panel had responses to both the visual stimulus and reward, the pause-rebound response being somewhat stronger for the stimulus than for reward. The neuron shown in the bottom panel responded selectively to reward.

**Figure 3:**
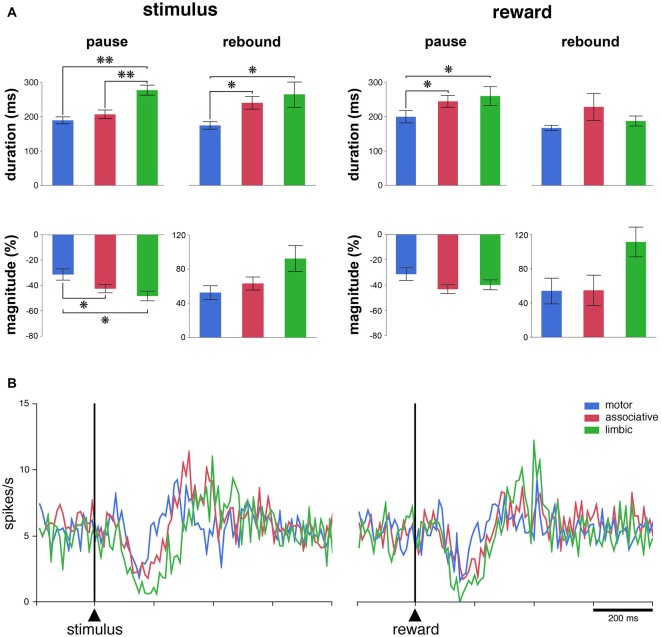
The responsiveness of TANs to task events in distinct striatal regions. **(A)** Comparison of durations and magnitudes of the two components of TAN responses (pause and rebound) to the stimulus and reward across striatal regions. Magnitudes of changes are indicated as decreases (pauses) or increases (rebounds) in percentage below baseline activity. Results are pooled for the two monkeys. Values are means ± SEM. **P < 0.01, *P < 0.05. **(B)** Population average activity of TANs recorded in the three striatal regions. Each curve indicates the mean activity averaged across neurons recorded in a given region for both animals as a function of time from stimulus onset (left) and reward delivery (right). Both the responsive and nonresponsive TANs are included.

To further examine how recording locations influence the responsiveness of TANs, we determined the durations and magnitudes of each of the two main components of the TAN response (i.e., pause and rebound) for each responsive TAN. For this analysis, data from the two monkeys were pooled. As shown in Figure 3A, the durations of pauses following stimulus onset were significantly different among the three striatal regions (*P* < 0.01, Kruskall-Wallis test), with the duration being longer in the limbic striatum than in the motor and associative striatum (*P* < 0.01, Wilcoxon test). Also, the magnitudes of pauses were significantly different among the three regions (*P* < 0.05, Kruskall-Wallis test), being higher in the limbic and associative striatum than in the motor striatum (Wilcoxon test, *P* < 0.05). An effect of region was equally seen on the durations of rebounds following the pause response to stimulus onset (*P* < 0.05, Kruskall-Wallis test), the durations being longer in the limbic and associative striatum than in the motor striatum (*P* < 0.05, Wilcoxon test). Although the magnitudes of rebounds did not vary significantly with respect to region (*P* > 0.05, Kruskall-Wallis test), there was a trend toward higher magnitude in the limbic striatum compared to the two other regions.

There were significant differences among the three striatal regions in the durations of pauses following reward delivery (*P* < 0.05, Kruskall-Wallis test), with the durations being higher in the limbic and associative striatum than in the motor striatum (*P* < 0.05, Wilcoxon test), whereas no significant effect of region was seen on the magnitudes of pauses (*P* > 0.05, Kruskall-Wallis test). The durations of rebounds following the pause response to reward did not vary significantly among the three striatal regions (*P* > 0.05, Kruskall-Wallis test) and the difference in the magnitudes of rebounds only approached significance (*P* = 0.053, Kruskall-Wallis test).

We next assessed the average activity of the entire population of TANs, regardless of whether or not the neurons were responsive to the stimulus and/or to reward. As shown in Figure 3B, there was a clear modulation of the whole sample of TANs recorded in each striatal region after each task event. The population activity aligned on stimulus onset diverged significantly from that in the control period at 110–120 ms after stimulus onset for the three striatal regions and the duration of the decrease in activity (i.e., pause) was 70, 110 and 160 ms in the motor, associative and limbic striatum, respectively. Therefore, the duration of the pause response to the stimulus in the limbic striatum appeared twice as long as in the motor striatum, confirming at the level of the population average that the pause response was longer in the ventral than in the dorsal striatal regions. In contrast, no differences could be observed in the duration of the increases in activity following the pause (i.e., rebound) across striatal regions. Also, a significant decrease in the population activity started 110–120 ms following the delivery of reward, with durations ranging from 70 ms in the motor striatum to 120 ms in the associative and ventral striatum. No significant increase in activity was detected, except in the limbic striatum. In summary, these results suggest a difference in TAN response features between the dorsal and ventral striatum, at the single neuron and population levels, after the reward-predicting stimulus and, to a lesser extent, after the reward itself.

**Figure 4:**
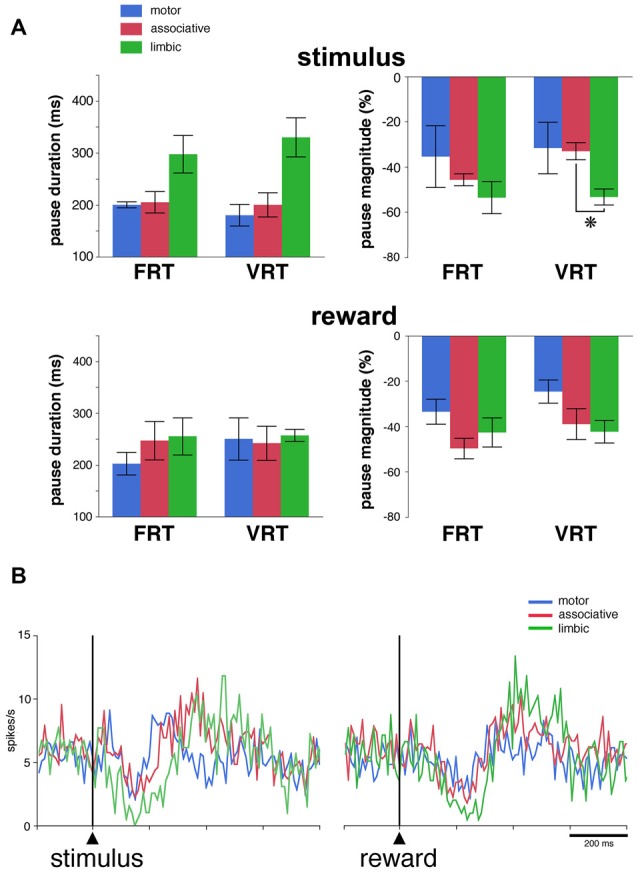
Pause responses of TANs in the VRT condition. **(A)** Effects of a variable stimulus-reward interval on the duration and the magnitude of TAN responses to the stimulus (top) and reward (bottom) in relation to striatal region. Results are pooled for the two monkeys. Values are means ± SEM. *P < 0.05. Same conventions as in Figure 3A. Numbers of neurons responsive to the stimulus and/or reward in the motor, associative and ventral striatum, are as follows: FRT, *n* = 5–7, 7–8 and 4–6, respectively; VRT, *n* = 4–6, 8–9, 5–6, respectively.** (B)** Population average activity across the 27 TANs recorded in the VRT condition. Same conventions as in Figure 3B.

We then determined whether regional-specific differences in TAN response features are influenced by changes in the length of the stimulus-reward interval. This was tested in 27 neurons (13 and 14 neurons in monkeys F and T, respectively) by replacing the fixed interval (FRT condition) by a random interval (VRT condition). Of these neurons, 17 (63%) responded to the visual stimulus and 20 (74%) to reward in the FRT condition and the fraction of neurons responding to the stimulus (17 of 27, 63%) or reward (21 of 27, 78%) was left broadly unchanged when tested in the VRT condition. The effects of the condition were assessed quantitatively by comparing durations and magnitudes of response for TANs which were responsive in both conditions. This was done only for the pauses, because the numbers of rebound were too small to perform a similar analysis. The resulting sample included 15 and 16 neurons with pause responses to the stimulus and reward, respectively. As shown in Figure 4A, we found that durations of pause responses to the stimulus only approached significance among the three striatal regions in the FRT and VRT conditions (*P* = 0.059 and 0.055, respectively, Kruskall-Wallis test). We also found that magnitudes of pause responses to the stimulus were significantly different in the VRT condition (*P* < 0.05, Kruskall-Wallis test), being higher in the limbic than in the associative striatum (*P* < 0.05, Wilcoxon test). No significant differences were observed in the magnitudes of responses in the FRT condition (*P* > 0.05, Kruskall-Wallis test). Durations and magnitudes of reward responses lacked significant differences among the three striatal regions in both conditions (*P* > 0.05, Kruskall-Wallis test). The sensitivity to stimulus and reward was also assessed by examining the activity averaged over all 27 TANs tested in the VRT condition (Figure 4B). The duration of the decrease in population activity following stimulus onset was 30, 80 and 120 ms in the motor, associative and limbic striatum, respectively. Durations of reward responses ranged from 50 ms in the motor striatum to 110 ms in the associative and limbic striatum. This confirms previous findings obtained in the FRT condition that the duration of the TAN pause response was the longest in the limbic striatum.

**Figure 5:**
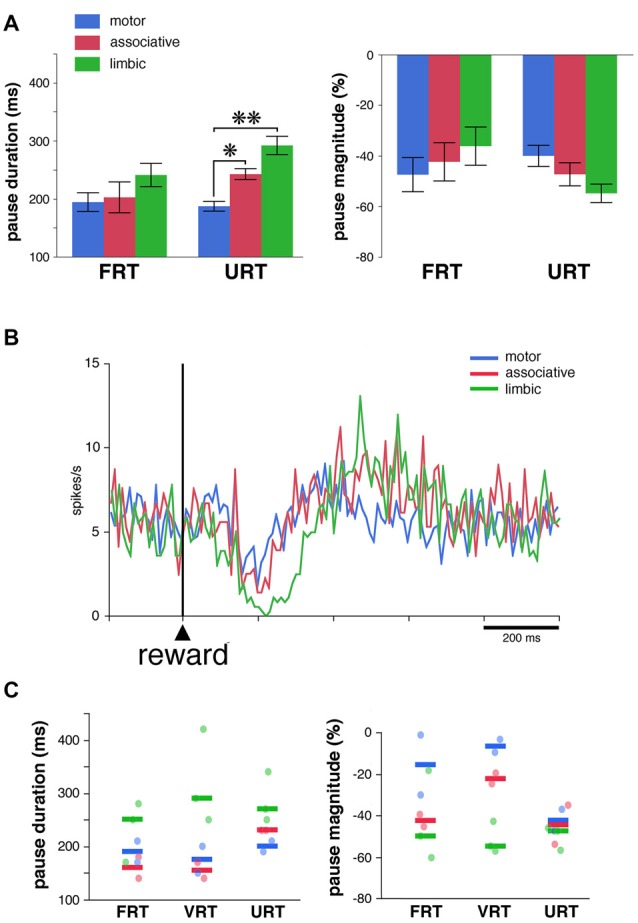
Pause responses of TANs in the URT condition. **(A)** Same conventions as in Figure 3A. Results are pooled for the two monkeys. Values are means ± SEM. *P < 0.05, **P < 0.01. Numbers of neurons responsive to reward in the motor, associative and ventral striatum, are as follows: FRT, *n* = 10, 5 and 11, respectively; URT, *n* = 10, 7, 10, respectively. **(B)** Population average activity across the 39 TANs recorded in the URT condition. Same conventions as in Figure 3B. **(C)** Response features of TANs as a function of striatal region in a sample of seven neurons tested in the three conditions. Each dot corresponds to a neuron responsive to reward and thick colored bars represent median values in different striatal regions.

We used the same analysis to investigate whether the absence of the visual stimulus predictive of reward influenced regional-specific differences in TAN response to reward. The activity of 39 TANs (22 and 17 neurons in monkeys F and T, respectively) was tested in the FRT and URT conditions. The proportion of TANs responsive to reward remained approximately the same when passing from the FRT (26 of 39, 67%) to the URT conditions (27 of 39, 69%). We further analyzed the responsiveness of TANs by comparing the durations and magnitudes of the pause response in 21 neurons which remained responsive to reward in both conditions (Figure 5A). As reported before, there were no significant differences in both the duration and magnitude of pause responses to reward among striatal regions in the FRT condition (*P* > 0.05, Kruskall-Wallis test). On the other hand, in the URT condition, duration of pause responses to reward were significantly different across striatal regions (*P* < 0.01, Kruskall-Wallis test), durations in the motor striatum being significantly shorter than durations in the limbic (*P* < 0.01, Wilcoxon test) and associative striatum (*P* < 0.05, Wilcoxon test). Also, magnitudes of pauses tended to be different among striatal regions (*P* = 0.07, Kruskall-Wallis test). Figure 5B represents the average activity of the whole sample of 39 TANs recorded in the URT condition. The duration of the decrease in activity was 70, 80 and 170 ms in the motor, associative and limbic striatum, respectively. It therefore appears that region-specific modulations of pause response were transferred from the stimulus predictive of reward to the reward itself when passing from the FRT to the URT.

Finally, a total of 14 TANs were each recorded for enough time to be tested in the three conditions, seven of them being responsive to reward in all conditions (3 and 4 in monkeys F and T, respectively). Even with this small sample size, a difference in the duration and magnitude of the pause response to reward was still visible depending on the striatal region (Figure 5C), the same neurons showing stronger responses in the limbic striatum as compared to the other striatal regions. This confirms the tendency for pause responses to be stronger in more ventrally located TANs. In summary, the pause response of TANs was stronger, in terms of duration and magnitude, in the limbic striatum compared to the other striatal regions, suggesting that differences in the functional properties of TANs may exist between the dorsal and ventral regions of the striatum.

## Discussion

Previous single-neuron recording studies in the striatum of behaving monkeys have established that TANs are prominently involved in the signaling and learning of motivational significance of stimuli (Apicella, 2002). Most of these studies have emphasized the constancy of response properties of TANs in the dorsal parts of the caudate nucleus and putamen, indicating that these neurons emit a signal that is uniformly distributed in the motor and associative regions of the primate striatum. On the other hand, no studies have been carried out in the ventral part of the striatum, also referred to as the limbic striatum, a region considered closely implicated in reward processing. The present study documents, for the first time in the monkey, the response properties of TANs located within the ventral striatum. Although we found TANs responsive to rewarding stimuli in all striatal regions explored, there were more frequent in ventral than in dorsal striatum. In addition, the duration of the typical pause response of TANs and, to a lesser extent, its magnitude were enhanced in the ventral striatum compared to the dorsal striatum. These region-specific differences in response features of TANs suggest that local cholinergic transmission may exert a differential influence on striatal circuitry which parallels functional specialization within the striatum along the dorsal/ventral axis.

### Functional Properties of Ventral TANs

We found that both individual TANs and the population as a whole showed different degrees of responsiveness to rewarding stimuli according to their location in dorsal or ventral regions of the striatum. Although the exact borders of the ventral striatum cannot be unambiguously delineated by electrophysiological recording, the caudal border of this region was operationally defined, as in our earlier study (Marche and Apicella, 2017), by the anterior commissure which serves as an anatomical landmark separating the anterior and posterior striatum in the primate. Ventral TANs that we sampled were located rostral to the anterior commissure and included the most ventral part of both the caudate nucleus and putamen based on depth readings on the microdrive.

As many studies have shown, we report that the dominant response of TANs to rewarding stimuli consisted of a pause in firing, often followed by a rebound activation. Of these two consecutive response components, the pause was more influenced by the recording site, being particularly strong in the ventral striatum in terms of duration and magnitude. Previous studies in our lab have reported that the pause in firing has less to do with the TAN’s contribution to reward prediction error coding than the subsequent rebound (Apicella et al., 2009, 2011), the early component showing relatively weak sensitivity to reward probability compared with that observed for the late component. However, despite the lack of statistically significant results in the present study, the rebound activation following the pause response to either the visual stimulus or reward tended to be more pronounced in the ventral striatum compared to the dorsal striatum. It therefore seems that both early and late response components of ventral TANs showed a trend toward being larger than those of the dorsal TANs. This heterogeneity in response features of TANs raises the possibility that cholinergic interneurons do not carry signals with homogeneous properties to dorsal and ventral striatal output pathways.

A few studies in behaving rodents have examined the activity of TANs in dorsal and ventral striatal regions. Although information on the neurons classified as TANs in rodents is not always in agreement with that collected in monkeys, some variation in TAN response properties has been observed between striatal regions (Yarom and Cohen, 2011). In particular, in rats trained to perform a nose-poke task, Benhamou et al. (2014) have reported that TANs in the dorsolateral striatum and ventral striatum were responsive to the stimulus that triggers the movement and to reward delivered after correct performance. Interestingly, these authors also noticed that pauses in firing in response to task events were approximately twice as long in the ventral striatum as in the dorsolateral striatum, an effect that is similar to the one described here in monkeys. Within the ventromedial striatum, a large proportion of TANs signal errors in the prediction of reward during unexpected delivery or omission of reward (Atallah et al., 2014) and it has yet to be examined whether this bidirectional outcome signaling is confined to the ventral striatum. However, there have been contradictory reports in behaving rats concerning the sensitivity of TANs to omission of expected reward (Benhamou et al., 2014). Thus, there is a need to further test whether TANs in the rodent striatum are able to indicate the presence or absence of rewards and whether these response properties are restricted to ventral TANs.

Other rodent inactivation studies argue that cholinergic interneurons in the dorsomedial striatum (rodent homolog of primate associative striatum) are closely tied to learning when behavioral flexibility is required (Ragozzino et al., 2009; Bradfield et al., 2013). Aoki et al. (2015) have compared the effects of selective lesions of striatal cholinergic interneurons in dorsomedial or ventral striatum on rats’ performance on a set-shifting task. Loss of cholinergic function in the dorsomedial striatum elicited impairments in set-shifting when changes in the action-outcome contingency require attention to a previously irrelevant stimulus, whereas loss of cholinergic function in the ventral striatum disrupted set-shifting when attention to a novel stimulus is required. These dissociable patterns of impairment in the rats’ ability to respond flexibly to environmental changes after striatal cholinergic damage may reflect differences in the impact of cholinergic transmission in ventral and dorsal striatal regions. In addition, selective optogenetic inactivation of cholinergic interneurons in the ventral striatum of freely moving mice, specifically the nucleus accumbens, has been reported to disrupt the expression of learned behaviors reinforced by cocaine (Witten et al., 2010).

### Influence of the Expected Time of Reward

In the FRT condition, we found that there were approximately equal numbers of TANs responding to the stimulus predictive of reward (68%) and to the reward itself (69%) across all striatal regions. This finding was somewhat unexpected, given the substantial evidence for reduced responsiveness of TANs to reward in Pavlovian and instrumental conditioning tasks in which the time of reinforcement is predicted by a stimulus and/or action (Sardo et al., 2000; Ravel et al., 2001). One explanation for this discrepancy might relate to differences in the levels of training. In the present study, the monkeys practiced the Pavlovian protocol only occasionally during recording sessions in which they completed hundreds of trials each day for many months in a probabilistic decision task in which the rewarding outcome was uncertain. It can be argued that extensive experience on this instrumental task might interfere with the development of an accurate prediction of the timing of reward under the Pavlovian conditioning procedure. We further demonstrated that the overall responsiveness of TANs to the stimulus and subsequent reward was not modified when we replaced the fixed 1 s stimulus-reward interval (FRT condition) by a randomly selected interval of 0.5, 1 and 1.5 s (VRT condition). As mentioned above, it is possible that the visual stimulus did not operate as a reliable predictor of the timing of reward, regardless of whether the interval is fixed or variable, due to the monkeys’ extensive experience under probabilistic schedules of reward in the instrumental task. Finally, although the fractions of TANs responding to reward were not different when reward was given in the presence (FRT condition) or absence (URT condition) of a preceding stimulus, interregional differences in response features were apparent when passing from one condition to the other. Namely, pause responses to the stimulus predictive of reward were strongest in the limbic striatum in the FRT condition, but without interregional differences in pause responses to reward, whereas pause responses to reward became strongest in the limbic striatum in the URT condition, as though differential TAN response features were shifted from the reward-predicting stimulus to the reward itself.

### Which Inputs Cause the Pause Response of TANs?

The interregional differences in response features of TANs that we document raise question about the specific inputs that drive this modulation. In particular, our findings suggest that the pause response in ventral TANs may originate from afferent sources that are distinct from those in dorsal TANs. The cellular mechanisms of the characteristic pause in cholinergic interneuron activity are still debated (Schulz and Reynolds, 2013). Striatal cholinergic interneurons receive afferent inputs from the cortex, thalamus and midbrain, and it is generally agreed that excitatory glutamatergic input from the intralaminar nuclei of the thalamus is the predominant input giving rise to the pause response (Reynolds et al., 2004; Ding et al., 2010; Doig et al., 2014). Anatomical studies also suggest that striatal cholinergic interneurons have a particularly strong relationship to the intralaminar thalamic nuclei in the monkey (Sidibé and Smith, 1999). The prevailing view is that the thalamic input drives initial excitation of cholinergic TANs, followed by an immediate pause in firing possibly resulting from an intrinsic membrane mechanism (Reynolds et al., 2004; Wilson and Goldberg, 2006). However, it has been repeatedly noticed that TAN pause responses may occur in the absence of an early brief excitation (Aosaki et al., 1994; Apicella et al., 1997), suggesting that the pause is not exclusively mediated by excitatory inputs to cholinergic interneurons. In this regard, midbrain afferents may directly participate in generating the multiphasic TAN response through corelease of DA and glutamate. *In vitro* studies using acute brain slice preparations from mice have shown that the response of striatal cholinergic interneurons induced by optogenetic activation of DA terminals may be directly driven by coordinated actions of DA and glutamate release acting on distinct receptor subtypes (Wieland et al., 2014). It has also been reported that the pause in the tonic firing of cholinergic interneurons triggered by optogenetic stimulation of DA midbrain projections in the dorsal and ventral striatum is dependent on regional variation in the corelease of glutamate and DA (Chuhma et al., 2014). There are other potential causes for changes in TAN firing. In particular, cholinergic interneurons receive inhibitory GABAergic inputs from axon collaterals of projection neurons in rodents and monkeys (Bolam et al., 1986; Gonzales et al., 2013). Such local interactions could permit striatal output pathways to regulate the expression of pauses in cholinergic TANs. Also, an inhibitory input from specialized populations of GABAergic striatal interneurons may contribute to the modulation of TANs (Szydlowski et al., 2013). In addition, phasic activation of GABAergic neurons of the ventral tegmental area that project to the ventral striatum (specifically, the nucleus accumbens) can directly inhibit the firing of cholinergic interneurons (Brown et al., 2012). It therefore appears that the source of the long-duration pause response of ventral TANs that we have observed may result from a combination of mechanisms. Determining the input types that are integrated by ventral and dorsal TANs to achieve their responsiveness to rewarding stimuli is a critical next step to understanding the specific contribution of local cholinergic innervation of the striatum to distinct aspects of behavioral performance.

### What is the Functional Meaning of the Pause in TAN Firing?

In primates, it is assumed that striatal TANs constitute a homogeneous neuronal population involved in the signaling and learning of the motivational significance of environmental stimuli. What is less clear in the current findings is what the specific information encoded in the duration of the TAN pause response and its consequence for behavior may be. We have previously provided evidence that a change in the temporal structure of the TAN response may serve as a neuronal mechanism for the discrimination of motivationally opposing stimuli (Ravel et al., 2003). It has also been reported that the modulation of response duration of neurons in basal ganglia structures, albeit not specifically striatal TANs, may carry information about probability of getting a reward (Parush et al., 2008). Recently, Franklin and Frank (2015) have pointed out that TANs may exert a powerful influence on behavioral adaptations at different levels of outcome uncertainty in a free-choice task. Based on computational simulations, these authors have postulated that the duration of the TAN pause is linked to action selection processes in a given learning situation. Namely, the pause is long for routinized actions generated in a stable environment, and short for actions requiring behavioral flexibility. Although it is difficult to assess whether this view is consistent with our behavioral conditions in which reward was given passively, it is possible that ventral TANs might have particularized responses in relation to a computation of stimulus-reward value that is important within the context of Pavlovian incentive learning. The ventral striatum is known to be important for the acquisition of stimulus–outcome relationships which can contribute importantly to various aspects of instrumental performance by signaling the occurrence and motivational value of behaviorally relevant stimuli (Balleine et al., 2009). Further studies are needed to clarify the exact role of the pause in TAN activity and how its duration relates to possible differences in postsynaptic processing of motivational information.

## Conclusion

Information processing in the ventral striatum is thought to play a role in mediating reward and motivation, whereas the dorsal striatum may be implicated in motor behavior and action selection. A description detailing the response properties of cholinergic TANs in these two regions may be important in determining the effect of acetylcholine on the output pathways of the striatum. In the present study we have observed that TANs display differences in their overall responsiveness to rewarding stimuli between ventral and dorsal striatum, with specific response features, which could be explained by the difference in the inputs they receive. Understanding the role of the cholinergic TAN circuitry in distinct striatal regions will provide insights into alterations in cholinergic signaling involved in the pathophysiology of multiple neuropsychiatric disorders.

## Author Contributions

PA designed the study. KM, A-CM and PA collected the data, analyzed the data, interpreted the data and wrote the manuscript.

## Conflict of Interest Statement

The authors declare that the research was conducted in the absence of any commercial or financial relationships that could be construed as a potential conflict of interest.
